# Supplementary material for: CNN-LSTM based emotion recognition using Chebyshev moment and K-fold validation with multi-library SVM
Source: PLoS One. 2025 Apr 7;20(4):e0320058. doi: 10.1371/journal.pone.0320058 (PMC11975114; doi:10.1371/journal.pone.0320058)
Supplement: Attachment — Submitted filename: Response to Reviewers.pdf [file pone.0320058.s001.pdf]

Reviewer 1:

Comment#1 Research Motivation:

Author Reply:

In page 6, The following contents are added

The motivation of this research study was to avoid unnecessary incidents i.e. could possibly be an accident due to driver's casualness. Though, so many existing models or approaches proposed thus far, this study was aimed to have a progress on this research field, considering the combinations of neural networks, deep learning methods and models. Driver's consciousness is very much important in ensuring driver's safety, also the pedestrians or fellow travelers. Thus, this study aims to achieve the same with following proposed combinations.

Comment#2Recent Works:

Author Reply:

In page 6, References 31, 32, 33, 34, 35 the following recent works are added

The authors have proposed face2face nodes based on novel graph model which does not require facial landmarks and this model had two components patch embedding with multi-scale feature fusion and relation-aware dynamic graph CNN. This model was mainly aimed to overcome the drawback of CNN's struggle to capture structural correlations and redundant correlation issue of Vision Transformers (ViT's). by incorporation K-nearest neighbors algorithm and relation-aware graph convolution operator, the model has achieved the following accuracies of 91.41%, 91.02%, and 66.69% on FERPlus, RAF-DB and AffectNet datasets, respectively.

In [31], a novel cross-hierarchy contrast (CHC) which employs contrastive learning and this model mainly regularizes feature learning and to improve global representation of facial images expression. A more robust feature representation was obtained using CHC and backbone network by integrating global features through fusion. The experiments were conducted on six widely used datasets, namely, CK+, FER2013, FER+, RAF-DB,

AffectNet and JAFFE. In [32], for accurate emotion detection from video clips, a novel cross attention and hybrid feature weighting network was proposed. This approach had three main components: Dual Branch Encoding (DBE), Hierarchical- attention Encoding (HAE) and Deep Fusion block (DF). In addition, recalibration block and adaptive attention block are integrated with HAE block which revises the feature map of each channel and to optimize fusion feature weights using hybrid weighting operation. Finally, DF block integrates all the features to predict the individual emotional state. The model was experimented on CAER-S dataset and results have shown that the model performed well on the video clips considered for this study.

In [33], the authors have proposed a CNN model with two approaches, namely, Transfer learning which is pre-trained with Inception-V3 and MobileNet-V2 and Taguchi method to improve the hyper parameter setting robustness. The experimentation was conducted of JAFFE and KDEF datasets with ten expression classes, where the proposed model has achieved an accuracy of 96% and F1-Score of 0.95%. In [34], DCNN model was proposed with pre-trained models like EfficientNet, ResNet and VGGNet along with Haar face classifier and the experiment was conducted on video streams unlike static images. The proposed model resulted in 82% accuracy on FER2013 dataset. In [35], Anti-Aliased Deep Convolution Network (AA-DCNN) was proposed to detect eight emotions from static image datasets like CK+, FER2013, JAFFE, and RAF. The main objective was to analyze how anti-aliasing can enhance expression recognition with down-sampling layers. This study has achieved highest accuracy on CK++ with 99.26%, and lowest with RAF at 82%.

Comment#3 Choice of Network Modules:

Author Reply:

In page 21, The following contents are added

This combination was proposed since CNN is very effective on extracting input image's local information from its previous layers and LSTM is effective in dealing with time-series of input sequence i.e. large or whole input sequence. The rich features obtained by CNN are fed to LSTM to determine or understand the dependencies that are relevant to the whole input image sequence. Transformer based techniques such as Vision Transformer (ViT) and Self Attention

Based SWIN transformer could be considered in our future research when dealing with cameras and videos to ensure safety of peoples who use road surfaces.

Comment#4 Chebyshev Moments:

Author Reply:

In page 9, The following contents are added

For the betterment of feature extraction, this study has considered Chebyshev moment which plays an important role as it has a repetition process to gain primary features from the input dataset images as this method is very much effective since it computes mean and standard deviation to determine where most of required image features or data fall within the image i.e. primary and secondary features.

Comment#5 Datasets and Generalization:

Author Reply:

In page 7, The following contents are added

Thus manual filtering was done on FER2013 dataset to overcome the above mentioned challenges, as a result, a total of 7074 images were selected based on contrast, brightness, angle of image. The selected images corresponds to five classes, namely, angry=966 images, fear=859 images, happy=2477 images, neutral=1466 images and sad=1326 images. Thus, out of 35000 images of FER2013 dataset, only 7074 were selected for this study based on study [13].

Comment#6 Ablation Study:

Author Reply:

Ablation study is not done in this work as we have considered neural network model and LSTM models along with other techniques that contributes to feature extraction and classification. While considering attention module or mechanism, ablation study could be performed to differentiate the values of performance metric with and without attention module.

For further research, this can be done to show the impact of adding or removing some modules on model's performance.

Comment#7 Image Quality:

Author Reply:

In page 15,16,17 The following contents are added

As per the comment, the quality of the figures 9,12,13,14 are improved.

Reviewer 2:

Comment#1

Authors have mistakenly written VGG9 in the graph and tables instead of VGG19. Kindly correct it.

Author Reply:

In page 14, 15, 16, The following contents are added

As per the above comment, VGG9 is changed into VGG19 on respective tables and graphs.

Comment#2

Could you please elaborate on the specific criteria used for the manual filtering of the FER2013 dataset? How were the factors such as contrast, brightness, and image angle quantitatively assessed during the process?

Author Reply:

In page 13 The following contents are added

The manual filtering is mainly performed to avoid images with occlusion, low quality and visual characteristics. Thus, the reduced dataset images will have only images with medium to high quality images that could lead to better detection of facial expressions as far as model's performance is concerned. As mentioned earlier in this paper, we used dataset from [13].

Comment#3

Why did you choose the Gaussian Filter and Chebyshev Moment for feature extraction? How do they compare to other commonly used feature extraction methods in the context of facial expression recognition?

In page 8, The following contents are added

Feature extraction is performed using Gaussian Filter to smooth out facial imperfections on images and automatic censoring was not performed with this filter as it reduces the image quality. which is followed by Chebyshev Moment to extract rich and primary features from the images based on the landmarks provided in Table 2. Chebyshev has a repetition process to gain primary features from the input dataset images as this method is very much effective since it computes mean and standard deviation to determine where most of required image features or data fall within the image i.e. primary and secondary features [37].

Gaussian filter is very effective in edge detection and also keep the edges relatively sharp which is also faster than median filter and also in removing the noise from the images. In case of box filter, the sharp cutoff leads to noisier output image with less well localized frequency response. Since, Gaussian filter create scale-space representation of an image with qualitatively reduced noise, object recognition can be enhanced [38].

Comment#4

In your training methodology, you combined CNN, LSTM and ResNet152. Could you provide more insights into how these architectures complement each other in this specific application? What advantages does the combined model offer compared to using CNN or ResNet152 alone?

Author Reply:

In page 10, The following contents are added

Architecture like CNN also suffers with such complexity images force the researcher to adapt such complex models like RENET50, RESNET102 and RESNET152 to overcome

performance degradation problem of CNN due to sensitivity and over fitting issues and challenges.

Resnet152 has much lower complexity and delivers better accuracy as it reduces 256 dimensions into 64 dimensions on 1*1 convolutions, then performs recovery through 1*1 convolutions again i.e. the process of both dimension reduction and dimension increase carried out. Figure 5 represents the RESNET152 with three layers deep adapted from [35].

Comment#5

What is the advantage of using Multi-library SVM classifier as the final classification step? How does it perform compared to traditional SVM or other classification methods like softmax?

Author Reply:

In page 13, The following contents are added

SVM is less prone to over-fitting problem as compared to other classifiers such as naïve base, but multi-class SVM contains separating hyper plane between the different classes of the target image is much easier and accurate in classifying the facial expressions.

Comment#6

Why did you manually select and filter the dataset? On what specific criteria were the images discarded, and what were the reasons for eliminating certain images?

Author Reply:

In page 13, The following contents are added

The manual filtering is mainly performed to avoid images with occlusion, low quality and visual characteristics. Thus, the reduced dataset images will have only images with medium to high quality images that could lead to better detection of facial expressions as far as model's performance is concerned. As mentioned earlier in this paper, we used dataset from [13].

Comment#7

Please improve the quality and symmetry of figures 9, 12, 13 and 14?

Author Reply:

In page 15, 16, 17, The following contents are added

As per the comment, the quality of the figures improved.

Comment#8

The purpose your paper is not clearly stated in the abstract. Please revise the abstract to indicate that the paper focuses on analyzing the driver's facial expressions to determine their mood or emotional state while driving. This will help readers to quickly understand the core objective of your study and its relevance to driver's behavior monitoring and safety.

Author Reply:

In page 1 The following contents are added

As per the comment, the abstract has been updated.

Comment#9

What is the connection between facial expressions and the driver's sleepiness? Are closed eyes considered as part of facial expression in your analysis, or are they treated separately? How do you address the relationship between expressions and drowsiness in your study?

Author Reply:

In page 13,14, The following contents are added

The drowsiness or closed eyes are not considered in this research work and with relevant datasets our future work might consider these expressions as well. The relationship between the facial expression and sleepiness should be an interesting scenario to study and it might impose some challenges as well.

Comment#10

Many references in the paper are outdated. Please update the references to include more recent source preferably from 2020 to 2024, to ensure that the research is aligned with the latest developments in the field.

Author Reply:

31. Fan Jiang, Qionghao Huang, Xiaoyong Mei, Quanlong Guan, Yaxin Tu, Weiqi Luo, Changqin Huang, Face2Nodes: Learning facial expression representations with relation-aware dynamic graph convolution networks, Information Sciences, Volume 649, 2023,119640,ISSN 0020-0255,https://doi.org/10.1016/j.ins.2023.119640.

32. Xuemei Wu, Jie He, Qionghao Huang, Changqin Huang, Jia Zhu, Xiaodi Huang, Hamido Fujita, FER-CHC: Facial expression recognition with cross-hierarchy contrast, Applied Soft Computing, Volume 145,2023,110530,ISSN 1568-4946,https://doi.org/10.1016/j.asoc.2023.110530.

33. Zhou, S.; Wu, X.; Jiang, F.; Huang , Q.; Huang, C. Emotion Recognition from Large-Scale Video Clips with Cross-Attention and Hybrid Feature Weighting Neural Networks. *Int. J. Environ. Res. Public Health* 2023, *20*, 1400. https://doi.org/10.3390/ijerph20021400.

34. Agung, E.S., Rifai, A.P. & Wijayanto, T. Image-based facial emotion recognition using convolutional neural network on emognition dataset. *Sci Rep* 14, 14429 (2024). https://doi.org/10.1038/s41598-024-65276-x.

35. Dhvanil Bhagat, Abhi Vakil, Rajeev Kumar Gupta, Abhijit Kumar, Facial Emotion Recognition (FER) using Convolutional Neural Network (CNN), Procedia Computer Science, Volume 235, 2024, Pages 2079-2089, ISSN 1877-0509, https://doi.org/10.1016/j.procs.2024.04.197.

36. Elsheikh, R.A., Mohamed, M.A., Abou-Taleb, A.M. *et al.* Improved facial emotion recognition model based on a novel deep convolutional structure. *Sci Rep* 14, 29050 (2024). https://doi.org/10.1038/s41598-024-79167-8.
